# Exploring Influential Factors Affecting the Severity of Urban Expressway Collisions: A Study Based on Collision Data

**DOI:** 10.3390/ijerph19148362

**Published:** 2022-07-08

**Authors:** Kun Wang, Xiaoyuan Feng, Hongbo Li, Yilong Ren

**Affiliations:** 1 School of Transportation Science and Engineering, Beihang University, Beijing 102206, China; fengxiaoyuan@scmif.com (X.F.); lihongbo@buaa.edu.cn (H.L.); yilongren@buaa.edu.cn (Y.R.); 2 Beihang Hangzhou Innovation Institute Yuhang, Hangzhou 310023, China

**Keywords:** traffic safety, urban expressway, collision severity, ordered logistic model

## Abstract

When traffic collisions occur on urban expressways, the consequences, including injuries, the loss of lives, and damage to properties, are more serious. However, the existing research on the severity of expressway traffic collisions has not been deeply explored. The purpose of this research was to investigate how various factors affect the severity of urban expressway collisions. The severity of urban expressway collisions was set as the dependent variable, which could be divided into three categories: slight collisions, severe collisions, and fatal collisions. Ten variables, including individual characteristics, collision characteristics, and road environment conditions, were selected as independent factors. Based on 975 valid urban expressway collisions, an ordered logistic regression model was established to evaluate the impacts of influence factors on the severity of these crashes. The results show that gender, collision modality, road pavement conditions, road surface conditions, and visibility are significant factors that affect the severity of urban expressway collisions. Females were more likely to be involved in more severe urban expressway collisions than males. For collisions involving pedestrians and non-motorized vehicles, the risk of more severe injury was 7.508 times higher than that associated with vehicle–vehicle collisions. The probability of more severe collisions on urban expressways with poor pavement conditions and wet surface conditions is greater than that on urban expressways with good pavement conditions and dry surface conditions. In addition, as visibility increases, the probability of more severe collisions on urban expressways gradually decreases. These results provide more effective strategies to reduce casualties as a result of urban expressway collisions.

## 1. Introduction

Urban expressways have become an increasingly important component in urban road systems. Compared with general urban roads, urban expressways have the characteristics of higher speed limits, dividers in the center of the road, the formation of three-dimensional intersections adopted at the intersection of roads, high road linear design requirements, and prohibited pedestrians, non-motor vehicles, and low-speed vehicles. They carry a large volume of vehicle traffic and play a pivotal role in serving medium- and long-distance travel and alleviating urban traffic congestion. Zhao et al. [1] found that the total length of second, third, and fourth ring road expressways accounts for only 8% of the length of the road network of Beijing but that they carry nearly 50% of the volumes of urban vehicle traffic. Similarly, urban expressways in Shanghai account for only 5% of the urban road area but they bear 35% of the city’s traffic. Maintaining a smooth and steady operation with respect to urban expressways is crucial to the overall the healthy operation of urban roads.

However, with the continuous increase in urban traffic demands, urban expressways have been in a gradual state of traffic overload in recent years. Heavy traffic volumes, high speeds, and frequent lane-changings are more likely to cause road traffic collisions, and compared with other roads, the severity of urban expressway collisions is often relatively serious. Traffic collisions not only aggravate traffic congestion in expressways but also may induce secondary accidents, further aggravating traffic congestion and the degree of casualties. It is of great significance to prevent the occurrence and reduce the severity of urban expressway collisions to ensure the safe and efficient operation of urban expressways. Therefore, this study aimed to identify the factors that significantly affect the severity of urban expressway collisions and then develop targeted preventive control measures to ensure travel safety on urban expressways.

A literature review on urban expressway traffic collisions is provided as follows.

To date, there have been a number of significant studies exploring the collisions that occur on urban expressways [2,3,4,5]. The literature on expressway collisions has shown that road user, collision, traffic flow, road, and environmental characteristics are the variables that are closely related to the incidence and severity of expressway collisions. In terms of road user characteristics, gender, age, and the driver’s experience were found to be variables that were strongly associated with the severity of injuries in expressway collisions [6,7]. Collision characteristics including the crash’s location, time, and season have been confirmed to have a significant correlation with the severity of injuries on expressways [8]. Collision modality has also been considered [9]. Hyodo and Todoroki [10] explored traffic collision factors on the risk of rear-ender collisions on urban expressways.

The influence of traffic flow characteristics on collision frequency and collision severity has also been extensively studied, with speed, speed variance, traffic volume, and acceleration being considered. Zhang et al. [9] found that the average speed, traffic volume, and speed limit have a significant impact on the frequency of crashes. Abdel-Aty found that the average speed is positively correlated with traffic collisions under high-speed conditions. Yu et al. [11] found that variations in volume and drops in speed increase the occurrence and likelihood of expressway collision during weekday peak hours.

In addition, the incidence rate and severity of expressway traffic collisions are also significantly different in different road environments [7]. Qu et al. [12] explored the risk impact of ramps on various types of locations across distinct traffic lanes (shoulder lane, middle lane, and median lane) and found that median lanes and sections after off-ramps have relatively lower risks compared to other lanes and sections. Fountas et al. [13] found that highway segments with long vertical curves (with lengths greater than the median of the corresponding vertical curve length distribution) and highway segments with wide shoulders (wider than 7.5 feet) are associated with injury outcomes of higher severity.

As traffic research is concerned not only with the prevention of traffic collisions but also with a reduction in the severity of traffic collisions, the severity of traffic collisions, a current major concern, has been widely studied in traffic collision research [14,15,16,17,18,19,20]. However, most studies have focused on freeway collisions or arterial collisions, and there are relatively few studies exploring the significant factors which affect the severity of expressway traffic collisions. Therefore, an ordered logistic regression model was developed to evaluate the impact of the factors that contribute to the severity of urban expressway collisions. The findings from this approach can provide a theoretical basis that supports traffic safety and decreases the severity of urban expressway collisions.

In the remainder of this paper, the data and study method are described in Section 2 and Section 3, respectively. In Section 4, the analysis results are presented. Finally, the implications of the study and its limitation are provided in Section 5 and Section 6, respectively.

## 2. Data

In this paper, the traffic collisions were obtained from a traffic collision data set for a Chinese city for the years 2014 to 2016. The data cleaning criteria were as follows: (a) variables that may have a significant influence were selected based on existing references and the collision data set, (b) collisions with missing records of these variables were excluded, and (c) the severity of the collision was defined according to the assessment of the body impairment of the injured in the road traffic collision. In total, 975 traffic collisions which occurred on urban expressways with complete records were ultimately obtained. These collisions resulted in 105 deaths and 1861 people were injured. The descriptive statistics for road traffic collisions on urban expressways are summarized in Table 1. Of the 1966 casualties involved in urban expressway collisions, 1744 (88.708% of the total) were male. Furthermore, of the 105 deaths involved in urban expressway collisions, 82 (78.095% of the total) were male. The average age of the casualties involved in urban expressway collisions was 36.369 years (S.D. = 9.907). In addition, although the 60 fatal traffic collisions which occurred on urban expressways only accounted for 6.2% of the total collisions, nearly two victims were involved in each fatal traffic collision. The present analysis is based solely on 975 traffic collisions on urban expressways.

To capture the potential influencing factors on the severity of traffic collisions on urban expressways, 10 variables, including individual characteristics, collision characteristics, and road environment conditions, were selected as independent factors according to existing research and the traffic collision data set. The casualty gender (coded 0 = male, 1 = female) was collected. The collision modality was divided into two groups (coded 0 = vehicle–pedestrian/cyclists collision, 1 = vehicle–vehicle collision), and the collision time was divided into two groups (coded 0 = daytime, 1 = nighttime). The road conditions included road pavement conditions, road surface conditions, road alignment, presence of roadside protection, and traffic signs and markings. The road pavement conditions were divided into two groups (coded 0 = good and 1 = bad), road surface conditions divided into two groups (coded 0 = dry and 1 = wet) and road alignment divided into two groups (coded 0 = linear section and 1 = nonlinear section). The variable for the presence of roadside protection was divided into two groups (coded 0 = no and 1 = yes). The presence of traffic signs and traffic markings was divided into two groups (coded 0 = complete and 1 = incomplete). The environment conditions included visibility and weather conditions. The visibility (unit: metre) was divided into four groups (coded 1 = less than 50, 2 = 50–100, 3 = 100–200, 4 = more than 200). The weather was divided into three groups (coded 1 = sunny, 2 = cloudy, 3 = rainy).

## 3. Methodology

In our analysis, the response variable for modeling the severity of collisions on urban expressways was the multinomial and ordered classification variable: slight collisions, severity collisions, and fatal collisions. Thus, an ordered logistic regression model was selected in this study to evaluate the impacts of influence factors on the severity of these collisions. The ordered multiple classification logistic regression model was extended from binominal logistic regression, which predicts an ordered multinomial dependent variable as a function of a series of predicting variables and has been widely used in traffic safety studies [21,22,23]. In the model, according to the ‘Assessment for body impairment of the injured in road traffic collision’ [24], the traffic collisions were divided into three categories: *Y* = 1 denotes slight collisions in which *i* traffic collision on an urban expressway involves slight injuries, *Y* = 2 denotes severity collisions in which *i* traffic collision on an urban expressway involves severe injuries, and *Y* = 3 denotes fatal collisions in which *i* traffic collision on an urban expressway involves deaths. As the probabilities of the dependent variables *Y* contain three categories, the two logit models were established with the 3rd category as the reference object can be written as follows:

(1) $$\mathrm{logit}\left(\frac{\pi_{1}}{1-\pi_{1}}\right)=\mathrm{logit}\left(\frac{\pi_{1}}{\pi_{2+}\pi_{3}}\right)=-b_{1}+\sum_{i=1}^{n}\alpha_{i}x_{i}$$

(2) $$\mathrm{logit}\left(\frac{\pi_{1}+\pi_{2}}{1-\pi_{1}-\pi_{2}}\right)=\mathrm{logit}\left(\frac{\pi_{1}+\pi_{2}}{\pi_{3}}\right)=-b_{2}+\sum_{i=1}^{n}\alpha_{i}x_{i}$$

where *P*(*Y* ≤ *j*) denote the probability of an incident being at or below a certain level of consequence and *π_i_* is the probabilities of the dependent variables *Y* containing three categories, respectively. $\alpha_{i}$ is the corresponding coefficient of the explanatory variable *x_i_*, and *b*_1_ and *b*_2_ are the intercepts of each regression model.

Most of the above influencing factors were dummy variables that needed a classification assignment, and the specific assignment did not represent the actual value. Among the above-mentioned influencing factors, visibility belonged to the multi-category dummy variable. The dummy variable was then transformed and assigned in the calculation process of the actual model: if the dummy variable had *k* categories, the dummy variable was converted to *k*−1 variables, and one of them was selected as the consultative variable. The conversion assignment of the visibility variable is shown in Table 2. The other variables were binary dummy variables, which were assigned 0 and 1 without the need for dummy variable conversion.

## 4. Results

### 4.1. Multicollinearity Diagnostics

To avoid the potentially serious influence of multicollinearity between the independent variables on the regression results, a multicollinearity test of the independent variables was required. The Model yielded values greater than 0.1 for tolerance and a value less than 5 for each variance inflation factor (VIF), which indicated that there was no potential multicollinearity among the selected independent variables. The results of such a multicollinearity test in Table 3 showed that the tolerance of each of the selected independent variables was much higher than 0.1 and that each variance inflation factor was less than 5. These results indicated that there was no potential multicollinearity among the selected independent variables, meaning that they could be used for an ordered logistic regression analysis.

### 4.2. Test of Parallel Lines

Ordered logistic regression analysis also requires the assumption of ‘proportional advantage’ that can be judged by the test of parallel lines. A significance level of 0.05 is usually the threshold for judging whether to reject the assumption of parallelism, and if the p value is greater than 0.05, this indicates that the regression equations are parallel to each other [25]. The result showed that the *p*-value was 0.707, which is much higher than 0.05, meaning that the ordered logistic regression model could be used for analysis.

### 4.3. Model Estimation

The ordered logistic regression model for traffic collision severity on urban expressways was constructed using the aforementioned methodology. The fatal collision variable was defined as the reference-dependent variable in this model. Table 4 lists the results of the modeling results.

The results in Table 4 show that four independent variables were found to be significant in the model estimates: gender, collision modality, collision time, road pavement conditions, road surface conditions and visibility. Male drivers were only 0.302 times more involved in severe injuries than female drivers on urban expressways. For collisions involving pedestrians and non-motorized vehicles, the risk of more severe injury was 7.508 times higher than for vehicle–vehicle collisions. Drivers in collisions on urban expressways with poor pavement conditions and wet surface conditions were 9.535 and 2.524 times, respectively, more likely to be more severely injured than drivers in collisions on urban expressways with good pavement conditions and dry surface conditions. As visibility increases, the probability of more severe collisions on urban expressways gradually decreases. In addition, the model intercept is 0.946 and 1.233 when all the independent variables are 0 (baseline state). This indicates that the incidence of slight collisions is 2.575 times higher than that of fatal collisions, and the incidence of non-fatal collisions (slight collision and severity collision) was 3.432 times that of fatal collisions. This also shows that there are a higher number of slight collisions on urban expressways than severe collisions and fatal collisions.

### 4.4. Model Validation

Goodness-of-fit statistics, such as the log-likelihood at zero, log-likelihood at convergence, Nagelkerke R2, and Akaike information criterion (AIC), were used to examine the degree of fit of the model, as shown in Table 4. The model selected five independent variables, so the degree of freedom was 4. Referring to the critical value table for the chi-square test, the chi-square threshold was 9.49 at a significance level of 0.05. In this paper, the chi-square value of the constructed model was 109.657, which was greater than 9.49, and the significance was 0.000, which was less than the significance threshold. This result means that the model had a good fit and was effective. In addition, the overall prediction accuracy of the research model is 92.205%, also indicating that the model fit is acceptable.

## 5. Discussion

### 5.1. Gender

Females were more likely to be more severely injured than male drivers on urban expressways, which is consistent with the result of previous studies [6,26,27]. Male drivers respond more quickly, and their acceptance abilities, operational abilities, and driving abilities were higher than the abilities of female drivers [28]. Compared to females, males drive more often, and with this comes more experience, and males are more attached to driving and feel more confident doing so [29,30]. Previous research has also shown that in crashes of equal severity, women are more likely than men to be injured or killed [31,32]. In addition, Segui-Gomez [33] also found that vehicle airbags cause additional injuries to drivers and passengers, especially females. Therefore, females’ driving skills and emergency response abilities should be improved, especially in high-speed driving situations. In addition, gender differences should be fully taken into account in designing vehicle safety devices to ensure that they can maximize and ensure the safety of travelers.

### 5.2. Collision Modality

The probability of a more severe collision between vehicle–pedestrian/cyclists is 7.508 times that of a vehicle–vehicle collision, which is consistent with the results of previous studies [34,35,36]. Pedestrians or cyclists are extremely vulnerable to injury or even death in the event of a collision because they lack protective equipment. Theofilatos et al. [37] also found that the lack of protection in powered-two-wheelers (PTWs) makes PTW occupants more likely to sustain severe injuries compared to car and heavy vehicle occupants. More attention and protection measures should therefore be implemented with respect to vulnerable pedestrians. In addition, the speed of urban expressways is fast, and pedestrians and two-wheeler users should be strictly prohibited from entering the main roads of expressways. The punitive measures for pedestrians and cyclists who illegally enter urban expressways should be strengthened.

### 5.3. Road Pavement and Road Surface Conditions

Both road pavement conditions and road surface conditions have a significant negative impact on the severity of traffic collisions on urban expressways, which is consistent with a previous study [38]. Drivers in collisions on urban expressways with poor pavement conditions were more likely to be more severely injured than drivers in collisions on urban expressways with good pavement conditions. As we know, drivers’ driving speeds are usually faster on urban expressways, and they typically have insufficient time to react when they observe poor pavement conditions ahead. At this time, they usually choose emergency braking or steering, which are more likely to cause a severe traffic collision [39]. Therefore, it is very necessary to carry out regular inspections and maintenance of urban expressway pavements. At the same time, when the road pavement is damaged, warning signs or facilities should be added in time to remind drivers to slow down.

The probability of traffic collisions on urban expressways resulting in more severe driver injuries on roads with wet road surface conditions was higher than that on roads with dry road surface conditions, and this result is consistent with the relevant research [38,40,41]. In fact, driving speed has the most dominant effect on injury severity, and fatal collisions occurred more frequently on roads with higher speed limits [42,43]. The braking distance on wet road surfaces increases, and the probability of losing control of the vehicle after emergency braking increases on wet roads. It is particularly important to ensure driving safety on wet roads by reducing road speed limits and setting up warning signs.

### 5.4. Visibility

Visibility has a significant positive impact on the severity of traffic collisions on urban expressways. As the visibility increases, the probability of more severity collisions on urban expressways gradually decreases, which is consistent with the results of a previous study [44]. Speed and speed variance are the two important indicators that affect the severity of collisions [45,46,47]. Drivers do not tend to slow down substantially until the visibility distance is drastically reduced by fog [48]. Once a collision occurs, high speeds do not provide drivers with enough time to react, resulting in more serious consequences. Gao et al. [49] found that the standard deviation of speeds under hazy weather conditions is 30% higher than that under clear weather conditions and the difference is significant at the 5% level. Therefore, under low-visibility conditions, a reduction in the speed limits can greatly reduce the severity of collisions on urban expressways. Warning drivers to maintain an adequate distance under low-visibility conditions also can be implemented on urban expressways.

## 6. Conclusions and Limitations

The severity of road traffic collisions has become an important factor in traffic safety research. Many studies have explored the factors which affect the severity of traffic collisions, but most of these studies focus on freeway collisions or arterial collisions. The severity of expressway traffic collisions has not been deeply explored. Therefore, as this study’s main contribution, we developed an ordered logistic regression model to explore the factors which affect the severity of urban expressway collisions. A comparison between the findings of this study and previous studies is provided in Table 5.

More specifically, the variables gender, collision modality, collision time, road pavement conditions, road surface conditions, and visibility were found to be significantly correlated with the injury severity of expressway collisions. This indicated that particular road users (e.g., pedestrians, cyclists, and motorcyclists) and traffic situations should be the focus of road safety interventions in the context urban expressways. The finding that vehicle–pedestrian/cyclist collisions lead to more severe injuries than vehicle–vehicle collisions is noteworthy. In China, neither pedestrians nor cyclists have the right to enter urban expressways, and even motorcycles are not allowed to drive on them in some cities. However, some pedestrians and cyclists choose to enter urban expressways illegally for convenience, especially electric cyclists and motorcyclists. Therefore, a stricter regulation that prohibits pedestrians/cyclists from entering urban expressways should be developed to reduce or prevent the occurrence of serious crashes on urban expressways in China. Taken together, these findings provide useful guidance for stricter regulations and more efficient technical countermeasures to reduce or prevent the occurrence of serious collisions on urban expressways in China.

This study also has several limitations. Although the ordered logistic modeling approach used in this paper is a traditional method used when addressing nonlinear problems, it is comprised of fixed parameters, which restrict the effects of explanatory variables so that they are the same across individual injury observations. While the reasons behind the occurrence of road traffic collisions can be complicated, it is not practical to incorporate all of the potential factors into the crash frequency/occurrence model. In addition, there may be unknown interactions between the observed factors, leading to the fact that the influence of each factor on the severity of different collisions is not fixed. The above two situations can cause the same influencing factor to have different effects on the severity of different collisions, which is collectively referred to as unobserved heterogeneity. Therefore, compared to the ordered logistic model, recent advanced applications in the form of random parameter and multi-state models are a feasible way to address the effect of unobserved heterogeneity and obtain more generalizable conclusions [19,38,51]. In addition, the collision data in this study was taken from a single city, and future research could analyze and model urban expressway traffic collisions in other cities to validate the findings of our study. More variables such as road user characteristics, vehicle attributes, traffic flow characteristics, collision sites, and other exposure variables should be also considered in the study of factors affecting the severity of urban expressway collisions.

## Figures and Tables

**Table 1:** Sample characteristics.

Attribute	Range	Traffic Collision	Percent of Traffic Collision (%)	Death	Percent of Death (%)
Gender	male	903	92.615	82	78.095
	female	72	7.385	23	21.905
Collision modality	vehicle–pedestrian/cyclist collision	101	10.359	39	37.143
	vehicle–vehicle collision	874	89.641	66	62.857
Collision time	daytime	622	63.795	41	39.048
	nighttime	353	36.205	64	60.952
Road pavement conditions	good	959	98.359	99	94.286
	bad	16	1.641	6	5.714
Road surface conditions	dry	887	90.974	85	80.952
	wet	88	9.026	20	19.048
Road alignment	linear section	810	83.077	85	80.952
	nonlinear section	165	16.923	20	19.048
Presence of roadside protection	no	173	17.744	12	11.429
	yes	802	82.256	93	88.571
Traffic sign and marking	complete	936	96.000	93	88.571
	incomplete	39	4.000	12	11.429
Visibility (meter)	<50	62	6.359	5	4.762
	50–100	113	11.590	31	29.524
	100–200	297	30.462	32	30.476
	>200	503	51.590	37	35.238
Weather	sunny	754	77.333	82	78.095
	cloudy	72	7.385	7	6.667
	rainy	149	15.282	16	15.238

**Table 2:** Example of coding profession variable.

Visibility Conditions (Meter)	Parameter Coding		
	Visibility 1 (<50)	Visibility 2 (50~100)	Visibility 3 (100~200)
Visibility 1 (<50)	0	0	0
Visibility 2 (50~100)	1	0	0
Visibility 3 (100~200)	0	1	0
Visibility 4 (>200)	0	0	1

**Table 3:** Result of multicollinearity test.

Attribute	Collinear Statistics		Attribute	Collinear Statistics	
	Tolerance	VIF		Tolerance	VIF
Gender	0.985	1.016	Road alignment	0.520	1.923
Collision modality	0.910	1.098	Presence of a roadside protection	0.542	1.846
Collision time	0.883	1.132	Traffic sign and marking	0.945	1.058
Road pavement conditions	0.959	1.043	Visibility	0.784	1.276
Road surface conditions	0.671	1.491	Weather	0.692	1.445

**Table 4:** Parameter estimation result.

Independent Variables	B	Exp (B)	Sig.	95% Confidence Interval	
				Lower	Upper
Gender (base: female)	−1.197	0.302	0.002	−1.938	−0.456
Collision modality (base: vehicle–vehicle collision)	2.016	7.508	<0.000	1.384	2.648
Collision time (base: nighttime)	−0.210	0.811	0.459	−0.767	0.346
Road pavement conditions (base: bad)	−2.255	0.105	<0.000	−3.479	−1.03
Road surface conditions (base: wet)	−0.926	0.396	0.009	−1.619	−0.233
Road alignment (base: nonlinear section)	0.935	2.547	0.067	−0.065	1.936
Presence of a roadside protection (base: yes)	−0.89	0.411	0.085	−1.902	0.122
Traffic sign and marking (base: incomplete)	0.957	2.604	0.156	−0.364	2.279
Visibility (base: >200 m. unit: meters)					
<50	1.535	4.641	0.005	0.455	2.615
50–100	1.282	3.604	0.001	0.492	2.071
100–200	0.813	2.255	0.013	0.168	1.458
Weather (base: rainy)					
Sunny	0.476	1.610	0.357	−0.538	1.490
Cloudy	−0.666	1.514	0.366	−2.111	0.779
Intercepts (base: fatal collision)					
Slight collisions	0.946	2.575	0.431	−1.409	3.302
Severity collisions	1.233	3.432	0.305	−1.124	3.59
**Fitting indexes**					
Log-likelihood at zero	603.880				
Log-likelihood at convergence	494.223				
Nagelkerke R2	0.230				
AIC	518.223				
Overall prediction accuracy	92.205%				

**Table 5:** Comparison between the findings of this study and previous studies.

Factor Attribute	This Study	Previous Studies on the Severity of Urban Expressway Collision	
Gender (reference: female)	−	−	Ye et al., 2021 [7]
Collision modality (reference: vehicle–vehicle collision)	+	Rarely attempted	
Road pavement conditions (reference: bad)	−	Rarely attempted	
Road surface conditions (reference: wet)	−	−	Lee and Li, 2014 [40]
		+	Zhu and Srinivasan, 2011 [50]
Visibility	−	−	Shi and Deng, 2019 [44]

Notes: + indicates that variables are positively correlated with the severity of traffic collisions; − indicates that variables are negatively correlated with the severity of traffic collisions.

## Data Availability

Data will be made available on reasonable request from the corresponding author.
